# δ-opioid Receptor, Microglia and Neuroinflammation

**DOI:** 10.14336/AD.2022.0912

**Published:** 2023-06-01

**Authors:** Yuan Xu, Ronghua Chen, Feng Zhi, Shiying Sheng, Leena Khiati, Yilin Yang, Ya Peng, Ying Xia

**Affiliations:** ^1^Clinical Medical Research Center, The Third Affiliated Hospital of Soochow University, Changzhou, Jiangsu, China. Departments of; ^2^Neurosurgery and; ^3^Neurology, The First People’s Hospital of Changzhou, Changzhou, Jiangsu, China.; ^4^Dubai Medical University, Dubai, The United Arab Emirates.; ^5^Shanghai Key Laboratory of Acupuncture Mechanism and Acupoint Function, Fudan University, Shanghai, China.

**Keywords:** δ-opioid receptor;, inflammation, microglia, oxidative stress, neuroprotection, neuropathological disease

## Abstract

Neuroinflammation underlies the pathophysiology of multiple age-related neurological disorders. Microglia, the resident immune cells of the central nervous system, are critically involved in neuroinflammatory regulation and neural survival. Modulating microglial activation is thus a promising approach to alleviate neuronal injury. Our serial studies have revealed a neuroprotective role of the δ-opioid receptor (DOR) in several acute and chronic cerebral injuries by regulating neuroinflammation and cellular oxidative stress. More recently, we found an endogenous mechanism for the inhibition of neuroinflammation is closely related to DOR’s modulation of microglia. Our recent studies showed that DOR activation could strongly protect neurons from hypoxia- and lipopolysaccharide (LPS)-induced injury by inhibiting microglial pro-inflammatory transformation, while knocking-down DOR or restraining DOR activity promoted microglia activation and the relevant inflammatory events with an aggravation of cell injury. This novel finding highlights a therapeutic potential of DOR in numerous age-related neurological disorders through the modulation of neuroinflammation by targeting microglia. This review summarized the current data regarding the role of microglia in neuroinflammation, oxidative stress, and age-related neurological diseases focusing on the pharmacological effects and signaling transduction of DOR in microglia.

## 1. Introduction

Neuroinflammation is a defensive response of the innate immune systems to intracellular and extracellular stimuli within the central nervous system (CNS) [1]. Its original aim is to protect and defend the cells against external insults. However, excessive, and prolonged inflammatory responses contribute to synaptic impairment, neuronal death, and even the onset and aggravation of several pathologies within the brain [2].

Microglia are the major effector of neuro-inflammation by serving as the native immune surveillance of the CNS. Microglia activation can be the first step of the cerebral response, and the different polarizations of microglia determine its detrimental or neuroprotective role played within the CNS. Microglia classification traditionally follows two phenotypes: the M1(classical phenotype), characterized by the production of a variety of pro-inflammatory cytokines and superoxide, and the M2 (alternative phenotype), described as a suppressor of inflammation [3]. However, with the advancement of single-cell sequencing technology, emerging evidence suggests that microglia have multiple reactive phenotypes in response to different cellular contexts and pathological stages [4-7], thus contributing to their complex action in cerebral diseases. A better understanding of microglial neuroinflammation and its modulation may help provide new therapeutical options for neurodegenerative disorders.

Opioid receptors belong to a G protein-coupled receptors (GPCRs) family with three key members: μ, δ, and κ opioid receptors (MOR, DOR, KOR) [8]. Although the most prominent tag of these opioid receptors is their modulation of pain signaling, the analgesic effects are primarily through activation of MOR [9]. Interestingly, DOR, being less important in pain control compared to MOR with a low abuse liability [9], shows a unique potential in neuroprotection and inflammatory regulation. DOR activation or overexpression can effectively protect the neurons against several injuries, including hypoxic/ischemic injury and neurodegenerative injury [10-18], and these protective effects are closely associated with DOR’s interaction with several intracellular compartments and regulation of inflammatory cytokines through mitogen-activated protein kinases (MAPKs), Nrf2 and PI3K/Akt pathways [19-21]. Indeed, DOR-mediated neuroprotection has been well documented in primary cultured neurons, brain slices, and animal brains in vivo [8]. An in-deep understanding of the multifunctional DOR and its underlying mechanisms may yield great potential for better prevention and treatment of neuroinflammatory injury in various neurological disorders.

Since microglia is a central player in neuroinflammation, we have recently explored the linkage between DOR and microglia and found that microglia are critically modulated by DOR-mediated signaling with a significant down-regulation of microglial neuroinflammation under stress [22]. Our novel finding suggests a therapeutic potential for neuroinflammatory injury in neurological disorders through a DOR-mediated modulation of microglial neuroinflammation. This review focuses on this new topic, with recent research progress regarding DOR’s distributions, functions, and signaling transduction involved in the modulation of microglial neuroinflammation.

## 2. Microglia are a major player in neuro-inflammation and oxidative stress

**Figure 1.**
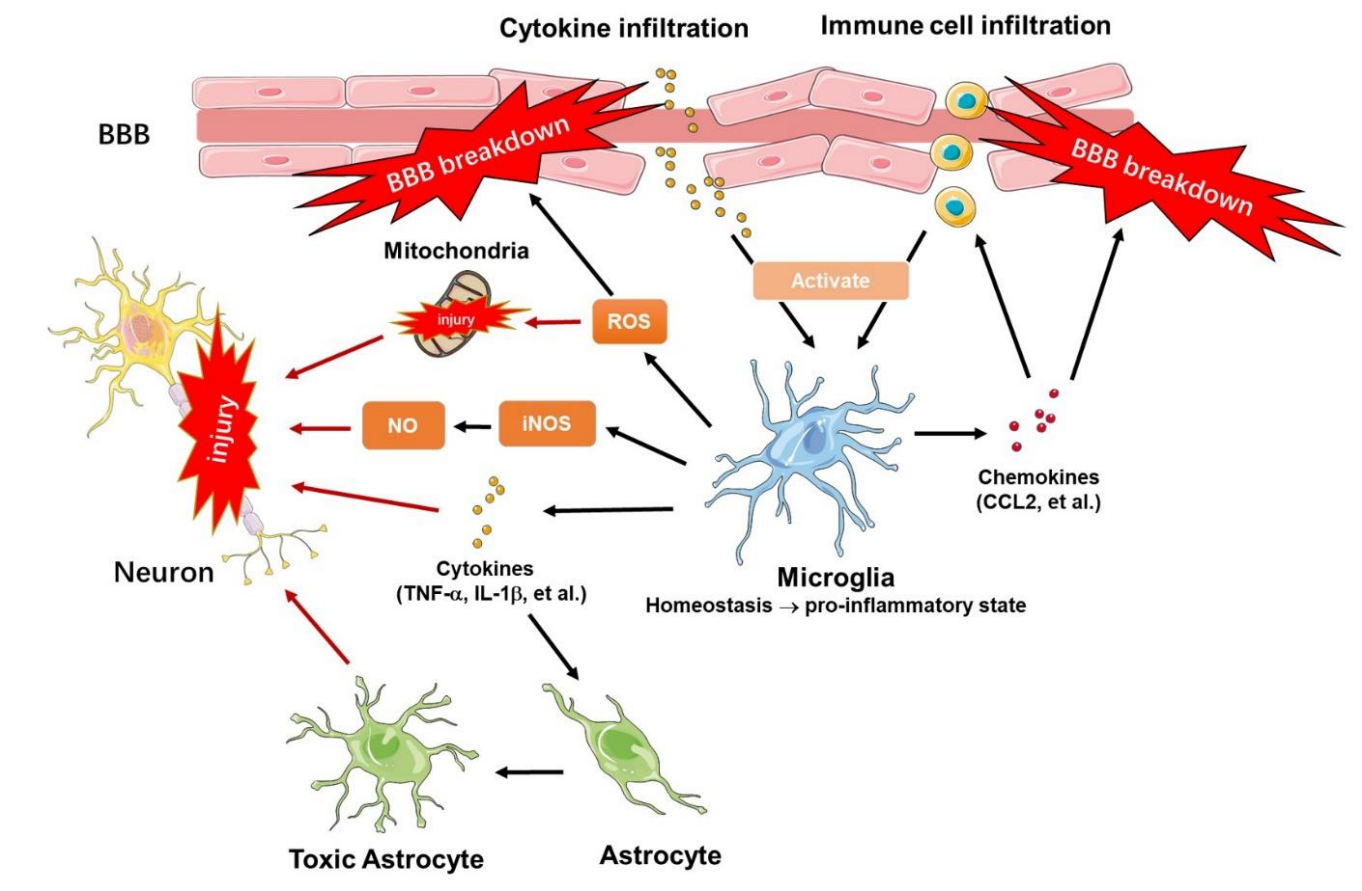
**Schematic of microglia-mediated neuroinflammation.** High levels of inflammatory cytokines and immune cells enter the brain by disrupting the tight junctions between different cell types within the brain vasculature of BBB, activating microglia toward a pro-inflammatory phenotype [132, 133]. Activated microglia produce various inflammatory mediators, including cytokines, ROS, iNOS, and chemokines [1]. An increase in inflammatory cytokines induced neuron apoptosis and aggravated the injury by converting astrocytes to toxic phenotypes [31, 32, 134]. Chemokines function as a chemoattractant for immune cells and molecular messengers in crosstalk among CNS- resident cells, which contributes to the disruption of BBB and the trafficking of immune cells to the brain [28]. Oxidative stress induced by ROS and iNOS is another enhancer for neuroinflammation that aggravates neuron injury. It can trigger inflammatory responses by activating several genes involved in the regulations of inflammatory cascades [29, 30]. The excessive production of ROS also led to mitochondrial dysfunction and tissue injury [135].

Microglia are the resident macrophages in the CNS, accounting for 10% of the total cerebral cells [3, 23]. The occurrence of neuroinflammation involves the participation of the blood-brain barrier (BBB), glia, and neurons. A microglia-mediated inflammatory response is the most common presentation in acute and chronic neuroinflammation [24-27]. Such as, lipopolysaccharide (LPS), an endotoxin that induces the pro-inflammatory response of microglia, can bind to the toll-like receptor 4 (TLR4) on the microglial surface, and promote the production of several pro-inflammatory cytokines, chemokines, and inducible enzymes. Cytokines such as TNF-α and IL-1βare critical initiators for neuronal apoptosis [1, 2]. Chemokines like CCL2 function as chemo-attractants to promote the crosstalk of immune cells and molecular messengers with CNS-resident cells, which contribute to the pro-inflammatory events in the CNS [28]. Reactive oxygen species (ROS) and inducible nitric oxide synthase (iNOS) are alternative enhancers for neuroinflammation. They can trigger pro-inflammatory responses by activating several inflammatory-related genes. Moreover, the excessive production of ROS disrupts mitochondrial integrity and leads to tissue damage [1]. The co-existence of iNOS and ROS generate more cytotoxic agents that have been implicated in neuron death [1, 29, 30] (Fig. 1).

The interplay of microglia with astrocytes is also involved in the neuroinflammatory process (Fig. 1) [31, 32]. In an LPS injection Csf1r^-/-^ mouse model, scientists from Stanford University identified a novel role of activated microglia in inducing the A1 phenotype of astrocytes through secreting IL-1α, TNF-α, and C1q. The A1 transformations of astrocytes further damaged neurons and oligodendrocytes by producing neurotoxins, which are abundantly expressed in neurodegenerative diseases and drive disease progression [33]. More recently, a study on NLY01, a potent glucagon-like peptide-1 receptor (GLP1R) agonist, also suggests a close relationship between astrocytes and activated microglia. Their results showed that compared to the microglia pro-inflammatory transformation induced effects on neurons alone under α-synuclein (αSYN) stress, the toxic A1 astrocytes converted by microglia activation amplified the death of dopaminergic neurons in the αSYN preformed fibril (αSYN PFF) mouse model and human dopaminergic neuronal cultures [34].

The crosstalk between microglia, BBB, neurons, and astrocytes constitutes a massive neuroinflammatory network with microglia at its forefront (Fig. 1). Previous studies simply classify activated microglia to the pro-inflammatory phenotype and anti-inflammatory phenotype, and the development of therapeutic agents focuses on inducing anti-inflammatory phenotype microglia or inhibiting the pro-inflammatory activation of microglia. However, this classification now is not fit enough to describe the heterogeneity of microglia. More reactive microglial populations with their distinct signatures have been identified in models of neuro-inflammation and neurodegeneration [35]. They cannot be simply attributed to “good” or “bad” microglial subsets in neuroinflammation. In fact, microglial-induced neuroinflammation and microglial phagocytosis can be a double-edged sword in cerebral disorders. Clarifying the detailed relevance of microglia to neuroinflammation and its related diseases is of great significance.

## 3. Microglia contribute to neurological patho-physiology through neuroinflammation

Neuroinflammation is implicated in acute and chronic neurological diseases, appearing as either a result or cause during the disease progression. Microglial dysfunction contributes to neuroinflammatory pathophysiology in various neurological diseases. The updated cognition of microglia’s contributions to major neurodegenerative diseases are summarized below.

### 3.1 Microglia in ischemic stroke

Microglia activation initiates within a few minutes in response to an ischemic stroke [36]. The activation levels of microglia reach a peak after 2-3 days of the stroke and remain for several weeks before gradually decreasing [37]. Previous studies suggested that mechanisms driving microglia activation are diverse in different ischemic regions. In the ischemic lesion core, microglia activation is mainly triggered by the excitotoxicity induced by ischemia. Meanwhile, in the petri-infarct regions, damage-associated molecular pattern molecules (DAMPs) are the main factor activating microglia [38, 39]. Once microglia are activated, they dynamically change their phenotype with distinct morphologies and altered gene expression patterns. Pro-inflammatory phenotype (M1-like) and anti-inflammatory phenotype (M2-like) are two inflammation-related microglial phenotypes studied most in ischemic stroke [40-43]. Hu et al. induced focal transient cerebral ischemia in mice for 1 hr and examined the microglia polarization at 1 to 14 days of reperfusion. The results showed that the neuroprotective M2-like phenotype is the predominant form of activated microglia at the early stages of ischemic stroke but began to decrease on day 7 and returned to pre-ischemia levels by day 14 after stroke. However, the levels of M1-like phenotype genes gradually increased from day 3 to day 14 after ischemia, with a peak on day 14 [44]. Emerging evidence confirm this microglia activation profile. A recent study analyzed the gene expression of microglia at 7 days after transient middle cerebral artery occlusion (MCAO) in a mouse model and found that although microglia displayed a mixture of phenotypes in vivo, activated microglia tended to gradually skew towards a pro-inflammatory phenotype [40]. The temporary changes in microglial phenotypes were also investigated on MCAO model rats. The 3-month and 12-month MCAO rats showed a rapid and persistent M2-like polarization of microglia, while only mild upregulations of M1-like microglia were investigated. Beyond the standard M1/M2 classification, numerous activated microglia showed phenotypes with a wide spectrum of functions, especially in pathways related to TLR2 and dietary fatty acids [41].

The microglia phenotype profile in ischemic stroke model rats is different from the observations in ischemic stroke model mice. The reason behind these evolving and complex patterns of microglia activation still needs to be identified, not only in cultured cells but also in a variety of animal species. The dynamic changes of microglia phenotype also imply the multi-faces of microglia in ischemic stroke. Besides the described dual functions of microglia in neuroinflammation, microglial activation is associated with phagocytosis, neurogenesis, angio-genesis, and synaptic remodeling, which remove the damaged cells and debris, promote the regeneration and repair after ischemic stroke, to be protective [45]. A vast number of clinical studies on ischemic stroke and experimental MCAO animal investigation showed microglia knocking out increased cerebral infarct size and detrimentally affected the animal’s outcome, suggesting that microglia play a protective role in the progression of ischemic stroke [46, 47]. However, there is also evidence showing that specific depletion of microglia reduced ischemic stroke injury by increasing anti-inflammatory factors and decreasing iNOS^+^ cells [48]. The divergences are probably due to the differences in the models involved in different studies, ages of animal used and the time point when the microglia are regulated.

### 3.2 Microglia in Alzheimer’s disease

AD is the most common cause of dementia and is characterized by extracellular accumulations of amyloid-beta (Aβ) plaques and intracellular formations of neurofibrillary tangles [49]. It has been observed that microglia migrate towards Aβ and cluster around these plaques in vitro and in vivo [50, 51]. Human genetic studies further point out the key role of microglia in AD pathogenesis with the evidence that the majority of the AD risk genes are selectively or preferentially expressed in microglia instead of other cell types in the brain [52].

The microglia-Aβ-dependent neuroinflammation is a major contributor to AD pathogenesis. Studies have shown that remarkable increased levels of pro-inflammatory cytokines and large amounts of activated microglia were detected in cerebrospinal fluid (CSF) and brains of AD patients compared to peers with no AD suffering [53, 54]. The inflammatory environment induced by activated microglia and astrocytes can increase β-site amyloid precursor protein cleaving enzyme 1 (BACE1) promoter activity and transcription and then up-regulate β-secretase cleavage and Aβ production [55]. Aβ in turn, activates Toll-like receptors (TLRs) and the NLRP3 inflammasome [52, 56], accelerating the pro-inflammatory conversions of microglia, which exacerbate AD neurodegenerations. It was reported that most microglia surrounding the Aβ plaques are activated with a protective phenotype at the beginning but later switch to the neurotoxic ones at the advanced stage of AD [4]. The key role of microglia-mediated neuroinflammation in AD is further highlighted by the close linkage between microglia and tau pathology. Laurent et al. summarized the vicious circle comprising microglia and tau [57]. Hyperphosphorylated tau triggers microglia activation and astrocytes toxic transformation, promoting pro-inflammatory cytokines and neurotoxic molecule production. These products, in turn, enhance tau pathology by modulating tau kinases such as p38 and cdk5. Activated microglia also released exosomal tau and promoted tau spread by phagocytosis of pathological tau [57].

More recently, single-cell sequencing technology has revealed a novel cluster form of microglia called as disease-associated microglia (DAM), which is activated in response to AD pathologies [50]. In an AD-transgenic mouse, the DAM program runs in a two-step process: initiation through a triggering receptor expressed on myeloid cells 2 (TREM2)-dependent stage and followed by a TREM2-independent program [35, 58]. Emerging evidence suggests a protective potential of such unique microglia-type in alleviating AD as this subset is featured by heightened expression of genes responsible for microglial chemotaxis and responses to Aβ plaques such as TREM2 and genes for phagocytosis such as Axl [50, 58, 59]. However, what is the relationship between DAM and neuroinflammation, and the alternations of inflammatory cytokines and chemokines in DAM have not been thoroughly studied. In-depth research will be needed to better understand these questions and DAM’s detailed effects in AD.

### 3.3 Microglia in Parkinson’s disease

PD is the second most common neurogenerative disorder after AD. Lewy bodies and dopaminergic (DA) neuronal death in the substantia nigra (SN) of the brain are the two most characteristic pathological hallmarks of PD [60]. Activated microglia with high expression levels of MHCII have been investigated in the SN and putamen of PD-affected brains [61] and microglial activation level correlates with dopamine terminal loss at the early stage of PD [62]. Bido et al. generated a novel mouse model with a selective αSYN accumulations in microglia [63]. They found that these αSYN mice developed progressive loss of DA neurons, one of the main features of Parkinsonism. The microglia stimulated by αSYN showed a large deprivation of phagocytosis and most of them transformed into a strong pro-inflammatory state [63].

One component that drives the interaction of αSYN with microglia is TLR. Previous studies suggest that the oligomeric αSYN activated microglia through heterodimer TLR1/2 [64]. TLR1/2 engaged at the cell membrane directly by αSYN and promoted the nuclear translocation of NF-κB, resulting in the microglia activation and pro-inflammatory cytokines release [64]. Activations of NLRP3 inflammasome is another potential pathogenic mechanism involved in the αSYN-microglia axis. It was reported that increased NLRP3 proteins were examined in PD models in vitro and in vivo [65, 66]. NLRP3 inflammasome activation can be triggered by fibrillar αSYN that drives dopaminergic neuropathology and neuroinflammation [67]. On the contrary, NLRP3 deficiency significantly reduced DA loss in MPTP- treated mice and attenuated microglia inflammatory activation with decreases production of IL1β [66].

NLRP3 inflammasome is also closely associated with impaired autophagy in microglia, which corporately contribute to the PD pathology. Evidence showed that microglia autophagy deficiency significantly activated NLRP3 inflammasome, then sequentially upregulated downstream IL-1β, and in turn increased the expression of macrophage migration inhibitory factor (MIF), a pro-inflammatory cytokine [68]. The neuroinflammatory events induced by the NLRP3 inflammasome further promoted the loss of hydroxylase (TH) neurons in the SN [68]. These findings were verified by a recent study [69]. In BV2 cells treated with LPS and PD mouse models, researchers found that the disruption of autophagy in microglia by Atg5 knockdown or knockout aggravated inflammatory responses and caused exacerbated neuron loss. These detrimental effects induced by microglia autophagy deficiency were majorly through modulations of NLRP3 inflammasome [69].

Cumulatively, these findings suggest that several age-related neurological disorders could be critically affected by microglia functional alternations and microglia-related neuroinflammation. Modulating microglial phenotype and preventing inflammatory events can be promising therapeutic strategies for these diseases.

## 4. δ-opioid receptors in neurons versus microglia

DOR is widely distributed in the CNS with a high expression level in the cortex, caudate, putamen, and amygdala [8, 70, 71]. Our previous studies have well demonstrated that DOR activation protects cells against various injuries: hypoxic/ischemic, parkinsonian, and Alzheimer’s [8, 13, 14, 16, 17]. Mechanistic studies showed that DOR-mediated neuroprotection is mainly through stabilizing ionic homeostasis, inhibiting excitatory transmitter release, and attenuating neurotransmitter disturbance in acute phase of stress, while DOR presents anti-oxidant capacity, inhibits inflammation, and regulates several signaling pathways: PI3K/Akt, extracellular signal-regulated kinase (ERK) and BDNF-TκkB to attenuate CNS injury during prolonged stress [11, 72-74]. However, most of these mechanistic investigations were conducted on neurons or neuron-like cells [10, 12, 13, 16, 17, 21, 75], but not other type of cerebral cells. Since microglia play a crucial role in CNS immune response and are involved in the regulation of cerebral homeostasis and neuron survival [76-79], it is interesting to explore whether DOR acts on microglia or even acts as a linkage between microglia and neurons. In this section, we compared the expression pattern of DOR in neurons versus microglia and summarized the current research progress regarding DOR-mediated signaling transduction in neurons versus microglia.

### 4.1 Neural DOR expression and intracellular signaling

Early in 1992, DOR was firstly identified from the neuroblastoma x glioma cell line NG-108 by two independent groups [80, 81]. After that, DOR was validated to express in neurons distributed in the cortex, spinal cord, and dorsal root ganglia (DRG) of mice, rats, turtles, monkeys, and humans [70, 82-84]. Erbs et al. reported that DOR is mainly present in GABAergic neurons, participating in the dynamic regulation of hippocampal activities [85]. Ma et al. [73] examined the changes in DOR expression in primary cultured rat cortical neurons and found that mild hypoxia caused an increase in DOR mRNA and protein, while severe hypoxia significantly decreased DOR expression with serious cell injury, suggesting DOR expression is sensitively influenced by environmental stresses [8, 73, 74].

DOR participates in a diverse array of neural activities [8, 86]. G protein-gated inwardly rectifying potassium (GIRK) channels have long been regarded as an essential component in the regulation of cell excitability in the brain [87]. Studies have demonstrated that DOR functionally interacts with the GIRK channels to maintain neuronal ionic homeostasis [8, 72, 88]. DOR activation by binding with ligands (UPF-512, DPDPE, SNC80, etc.) caused a conformational rearrangement between DOR-Gαo and DOR-Gβγ. The conformational alternations in Gβγ then induce the conformational changes at the Gβγ/GIRK interface to increase channel permeability and evoke channel current in the neuronal membrane [89]. DOR also interplays with transporters and receptors to regulate neurotransmitter release. Its direct interactions with glutamate transporters, GABA transporters, dopamine transporters, N-Methyl-D-Aspartate receptors (NMDARs), GABA_A_ receptors, and dopamine D receptors are well documented in previous studies [8].

DOR targets serial signaling cascades in neurons. As a family member of GPCR, DOR regulates ERK/p38 MAPK and PI3K/Akt signaling in a GPCR-dependent manner by stimulating protein kinase C (PKC) in neurons [72]. Studies also showed that DOR signaling are closely associated with other important pathways including Caspases, NF-κB and Nrf2. Whether these pathways are activated through G protein signaling needs to be clarified [8]. Besides that, DOR can interact with different cellular compartments and largely affects several critical neuronal organelles [8, 72]. DOR activation contributes to synaptic improvement against global ischemia, as noted in a rat model [90]. Namely, the beneficial changes were induced by DOR on synaptic morphology and synaptic transmission through PKCα/MARCKS and BDNF/ ERK/synapsin I pathways [90]. Mitochondria provide energy for neuron survival by producing ATP. Mitochondria deficiency is implicated in the pathogenesis of stroke and neurodegenerative diseases [91, 92]. Our previous studies have revealed that DOR activation effectively attenuates mitochondrial respiratory chain injury by modulating Akt, Caspase, and ERK signaling pathways [13, 93], and DOR enhanced mitophagy in a PINK1-Parkin dependent manner under 1-Methyl-4-phenylpyridinium (MPP^+^) stress [16]. In highly differentiated PC12 cells and primary trigeminal ganglion neurons, Shiwarski et al. [94] investigated DOR regulated by Golgi export checkpoint PI3K C2A. The inhibition of PI3K activity or depletion of PI3K C2A blocked the delivery of newly synthesized DOR from the Golgi to the cell surface [94]. Evidence has also shown DOR-endoplasmic reticulum (ER) interactions in PD. In dopaminergic SH-SY5Y cells subjected to ER stress, Moghal et al. [95] found that DOR agonist DADLE treatment enhanced cell survival, downregulated UPR stress sensors, and prevented protein aggregation against ER stress. The mechanism is associated with the crosstalk between DOR and pro-survival MAPK-NGF-Bcl2 signaling pathways [95].

### 4.2. Microglial DOR expression and intracellular signaling

Although microglia have been demonstrated to express a wide range of GPCRs, including metabotropic glutamate receptors, purinergic receptors, and adenosine receptors [96], the expression and distribution of opioid receptors, especially DOR in microglia, have been less examined, and the findings are contradictory. An early study examined the cell-type-specific gene expression in the nucleus accumbens of rats and reported that DOR mRNA was undetectable by qPCR both in microglia and astrocytes in this brain region [97]. Mika et al. obtained similar results by detecting opioid receptor expression in rat primary cultured microglia. They found the mRNAs and proteins of MOR and KOR in microglia but not for DOR [98]. The first evidence validating the existence of DOR in microglia is provided by Shrivastava et al. They investigated the colocalization of DOR and microglia in the mice hypothalamus by using double immuno-histochemistry methods. The images showed an evident colocalization of DOR with iba-1 positive cells, suggesting microglia express DOR proteins in mice hypothalamus [99]. Moreover, in this study, microglia isolated from the neonatal rat hypothalamus were used to grow as primary cultures. Significant amounts of DOR were also detected in the proteins collected from primary cultured microglia, and DOR density increased after the cells were treated with a DOR agonist, DPDPE [99]. The expression of DOR in microglia was further validated in normal human and mouse brains by western blot analysis and immunofluorescence staining [100]. It is reported that LPS (0.5 μg/ml) induced the M1-like activation of microglia and strikingly upregulated DOR expression in vitro [100]. More recently, our group has also detected a mild expression of DOR in BV2 cells, an immortalized microglia cell line. A major difference in our experiments from the earlier studies is that we found DOR expression is positively associated with microglia M2-like transformation with the evidence that IL4 and hypoxia significantly increased DOR in BV2 cells, while LPS induced an inappreciable effect, and even caused a decrease in DOR expression in BV2 cells [22].

Compared to neuronal DOR signaling, microglial DOR signaling is less examined in previous studies as well. Broadly speaking, microglia is a primary participant in pain development. Microarray studies have revealed that microglia present dramatical changes in morphology, gene expression, proliferative potential, and function in neuropathic pain states [101, 102]. Since opioid receptors are an important regulator for neuropathic pain expressed in microglia [103, 104], it has been documented that opioid receptor activation conducts molecular signaling in microglia through many intracellular pathways, including ERK1/2, p38, NF-κB, PI3K/Akt, and etc. [105]. However, these studies focus on the interactions between opioids/opioid receptors and microglia in modulating neuropathic pain, but not other functions. Moreover, there is little data regarding DOR-mediated neuroprotective signaling transduction in microglia. Several fundamental questions remain unanswered. For example, does DOR drive the same signaling cascade in the microglia versus neurons? Does DOR-mediated signaling cascade affect microglia morphology, activation, function, and its crosstalk with other nerve cells? Clarifying the details of DOR action in microglia at cellular and molecular levels is of great significance for understanding the mechanisms of DOR-regulation of microglial activity.

## 5. DOR attenuates microglia-mediated neuro-inflammation

Clues point out the direct or indirect involvement of DOR in microglia-mediated neuroinflammation. Microglia polarization determines the state of microglia to be pro-inflammatory or anti-inflammatory. Our recent studies and those of others have provided strong evidence that specifically activating DOR could largely inhibit LPS, ethanol, or hypoxia-induced microglia pro-inflammatory activation in vivo and in vitro [22, 99, 100], while knocking-down DOR in BV2 cells promoted BV2 excessive activation [22]. An important aspect of microglia to amplify neuroinflammation is the release of pro-inflammatory cytokines and superoxide. Shrivastava et al. reported that DOR agonist DPDPE increased the basal level of anti-inflammatory cytokines in the primary cultured microglia and effectively prevented ethanol’s stimulatory actions on pro-inflammatory cytokines [99]. Studies from our laboratory and Cheng’s laboratory also showed that activated or overexpressed DOR in microglia inhibited the production of pro-inflammatory cytokines, including TNF-α, IL-1β, and IL6 under LPS and hypoxic insults [22, 100], while inhibiting DOR activity or knocking-down DOR expression largely increased the release of inflammatory cytokines and iNOS, which detrimentally affected the neurons in the microglial immune microenvironment [22]. Moreover, DOR activation could effectively attenuate iNOS and ROS release under several insults, protecting mitochondria integrity against injuries [16, 100, 106, 107].

DOR regulates neuroinflammatory processes at multiple levels and is closely associated with microglia. Karkischenko reported leytragin, a DOR agonist, could significantly inhibit high mobility group box 1 (HMGB1) secretion in LPS model mice [108]. Since HMGB1 has been identified to play an essential role in inflammatory responses by binding primarily to TLR4 and RAGE, stimulating microglia activation, DOR seems to be a promising therapeutic target for anti-microglia activation [108]. Autophagy is responsible for cellular quality control by selectively degrading damaged organelles and misfolded proteins. Evidence suggests that autophagy is activated in microglia and regulates microglial phenotype [109, 110]. The inhibitions of autophagic flux were reported to cause transformations of BV2 cells toward M1-like phenotype with increased release of TNF-α and iNOS and decreased M2-like markers: IL10, Arginase, and BDNF [110]. In our previous work, we have strongly demonstrated that DOR activation enhanced autophagy in Parkinsonian injury [16]. Consistently, DOR is likely to regulate the inflammatory activation of microglia through modulating autophagy.

Apart from that, microglial phagocytosis is a critical component of microglia immune response to CNS injury. Microglia remove impaired or degenerating neurons and axons to prevent the release of pro-inflammatory components and maintain a favorable environment for CNS restoration. Meanwhile, excessive microglia phagocytosis may accelerate neuronal loss and exacerbate neuroinflammation [111]. It is reported that morphine attenuated excessive phagocytosis of mouse microglia under LPS and interferon-γ insults, and naltrindole totally abolished the morphine effect [112]. Since morphine can induce opioid receptors activation: MOR and DOR, and only naltrindole inhibited the morphine’s effects on microglial phagocytosis, this study suggested the involvement of DOR in microglial phagocytosis during neuroinflammation.

## 6. Major pathways for DOR modulation of microglial neuroinflammation

**Figure 2.**
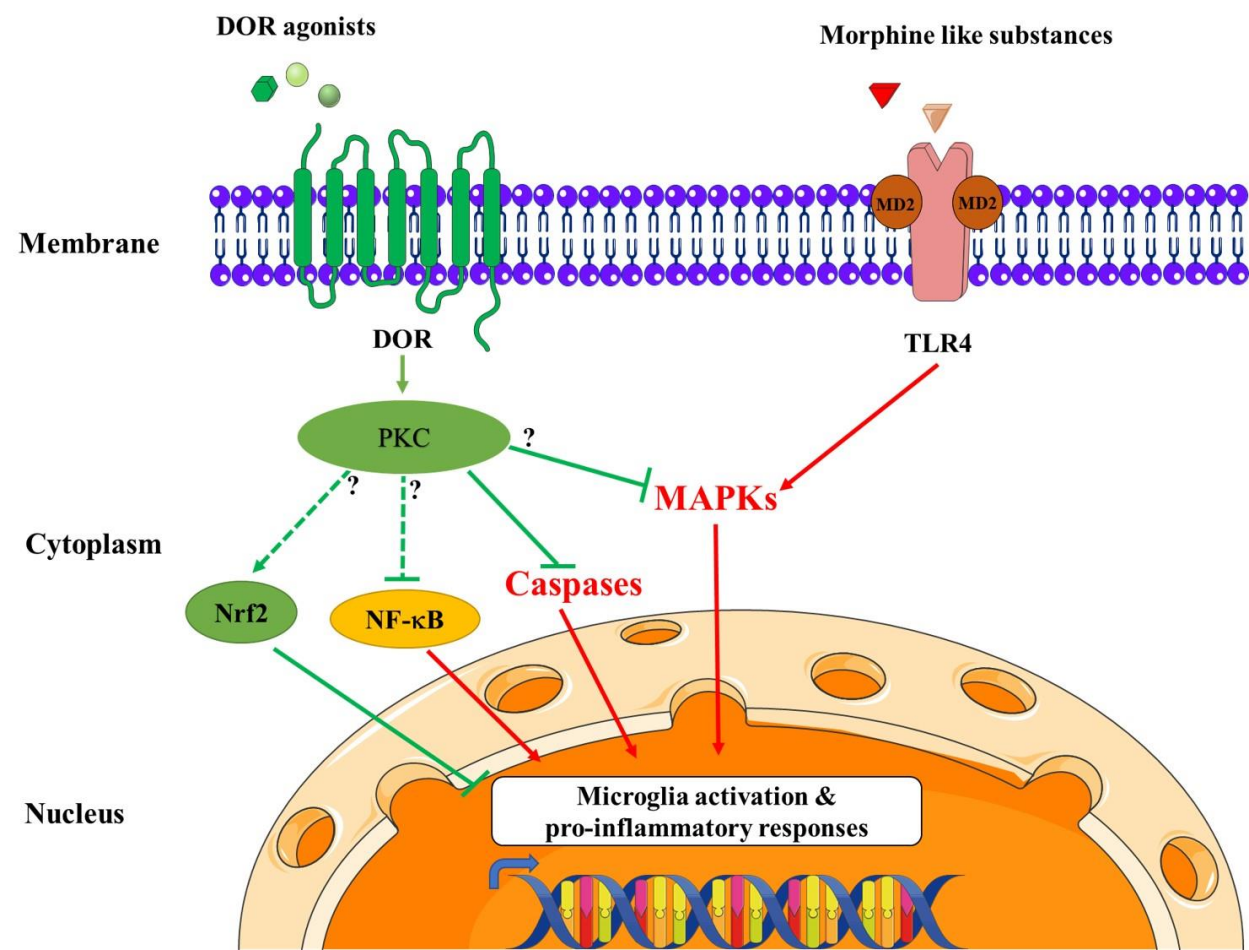
**Molecular mechanism underlying DOR-mediated signaling in microglia.** Opioids such as morphine tend to bind with the innate immune receptor TLR4, instead of MOR and DOR [37] and TLR4 forms a complex with Myeloid differentiation factor 2 (MD2), which induced the phosphorylation of MAPKs that activated microglia and its inflammatory responses to stress [100]. DOR agonists like UFP-512, DADLE, and SNC-121 can inhibit microglial activation and its related inflammatory events by direct actions on DOR. The potential pathway crosstalk between DOR and microglia includes MAPKs, Caspases, NF-κB, and Nrf2 [100, 122, 125, 127]. There is evidence showing that caspases regulated microglia activation through a PKC-δ dependent way in microglia [131]. However, whether other microglial DOR signaling goes through GPCR pathway, like in neurons, needs further experimental verification. Therefore, the question marks are placed on Nrf2, NF-κB and MAPK pathways since there is lack of direct data at present to demonstrate that DOR activates them through PKC pathway in microglia. The dotted arrows implies that there is no direct evidence showing DOR modulation of microglial activation via Nrf2 and NF-κB though neuronal studies have shown so.

There is limited documentation on the DOR signaling transduction in microglia. Traditional notions and recent findings showed that the activation of DOR is immunosuppressive and can inhibit the excessive activation and the pro-inflammatory responses of microglia to environmental stimuli [22, 99, 100]. However, strong evidence suggests that opioids such as morphine and fentanyl always induce activation of microglia rather than suppressing it [113, 114]. One possible reason for this inconsistency is that opioids have different affinities to different opioid receptors. For example, morphine has a much better binding to MOR than DOR with previous studies implying that MOR always showed contrary role from DOR in several pathogeneses and cell types [17, 99]. Another explanation is that opioids’ stimulatory effects on the immune system are independent of opioid receptors. Substantial clues indicated that TLR4 is a potential binding site for opioids. Opioids can bind and activate TLR4. TLR4 subsequently forms a complex with Myeloid differentiation factor 2 (MD2), stimulating its downstream signaling: MAPK [115, 116] (Figure 2). Genetic depletion of TLR4 blocks these effects [117, 118]. These findings were further validated by the observations that morphine-3-glucuronide (M3G), a morphine metabolite without opioid receptor activity, can bind and activate TLR4, but morphine-6-glucuronide (M6G), with active opioid receptor activity, cannot [116, 119].

### 6.1 MAPK

An essential participant of microglial inflammatory response is MAPK. The MAPK family consists of three members: ERK, c-Jun N-terminal kinase (JNK), and p38. Recent studies have revealed the importance of MAPK signaling in microglia-mediated neuroinflammation. Plastira et al. reported that administrations of MAPK antagonists attenuated lysophosphatidic acid (LPA), induced production of TNF-α and IL-1β, and reduced the microglia phenotype markers CD86 and CD206 [120]. Consistent with this finding, He et al. found that LPS triggered p38α MAPK-dependent phosphorylation of ULK1 in microglia and contributed to the reduced autophagy and increased production of IL-1β in microglia in vitro and in vivo [121]. Several agents were discovered to modulate inflammatory responses and inflammatory activation of microglia by targeting MAPK signaling, with the DOR agonist as a potential one. Cheng et al. have provided direct evidence that activating DOR using the DOR agonist Tan-67 significantly reduced the phosphorylation of ERK, JNK, and p38 in BV2 microglial cells and thus protected cells from LPS-induced inflammation and apoptosis [100]. DOR is also involved in the regulation of MAPK in chronic inflammation and parkinsonian injury. Administrations of DOR-specific agonist UFP-512 significantly decreased JNK and ERK phosphorylation under chronic inflammation while enhanced phosphorylated-ERK under acute MPP^+^ insult and hypoxic insult [13, 19]. Moreover, evidence also shows that morphine decreased LPS and interferon-γ induced microglia engulfment, while the addition of naltrindole, a selective DOR antagonist, attenuated this effect by promoting p38 phosphorylation [112].

### 6.2 NF-κB.

NF-κB has been identified to be activated in response to stresses and induce neuroinflammation in the brain. Many cytokines: IL-1, IL-6, TNF-α, chemokines, and monocyte chemoattractant protein 1 (MCP-1), are regulated by NF-κB [122]. In microglia, receptors on the cell membrane detect and bind with foreign antigens, activating NF-κB signaling. Activated NF-κB is then translocated to the nucleus, promoting microglia activation and pro-inflammatory cytokines release [42, 123, 124]. In a model of amyotrophic lateral sclerosis (ALS), NF-κB was selectively inhibited in astrocytes and microglia respectively, and the researchers found that only restraining NF-κB signaling in microglia rescued motor neuron death by impairing proinflammatory microglial activation [123]. After that, Zusso et al. reported that LPS exposure caused p65 subunit translocation from the cytosol to the nucleus as an indicator of NF-κB activation in microglia. Following this process, the concentration of IL-1β and TNF-α sharply increased [124]. The modulatory effects of DOR on NF-κB were also examined in rat and human immune cells. An early study demonstrated that activating DOR using DOR agonist SNC-121 attenuated TNF-α induced matrix metalloproteinase-2 (MMP-2) secretion in the glia cells, and the effects of DOR were realized by negatively regulating the p38 MAPK and NF-κB signaling [125]. More recently, a group of researchers established a MCAO rat model, in which cerebral ischemia-reperfusion injury increased TLR4 and NF-κB expression in the rat’s brain, which was remarkably inhibited by DOR-specific agonist DADLE [126]. NF-κB was regarded as one of the crucial signaling targets in DOR-mediated long-term neuroprotection. Zhu et al. reported the varying mechanisms underlying DOR-mediated short-term and long-term neuroprotection [72, 127]. Short-term DADLE stimulation protected neurons majorly through the PKC-mitochondrial ERK pathway, while sustained DADLE stimulation promoted Bcl-2 expression and elicited neuroprotective effects in the NaN_3_ model by targeting PI3K/Akt/NF-κB pathways [127]. Since there is no direct evidence proving DOR regulates NF-κB in microglia, further studies are needed to clarify the cascade signaling induced by DOR in microglia and its linkage with neuroinflammation.

### 6.3 Nrf2.

Nrf2 is an important player in anti-inflammation and anti-oxidation. Sulforaphane (SFN), a potent Nrf2 activator, increased Nrf2 activity and its downstream protein heme oxygenase-1 (HO-1) in LPS-treated BV2 cells. The activation of Nrf2/HO-1 signaling further downregulated the production of inflammatory mediators (iNOS, NO, and COX-2) and pro-inflammatory cytokines (TNF-α, IL6, and IL-1β) in microglia exposed to LPS [128]. Nrf2/HO-1 induced microglia modulations are validated in animal models. In spinal cord injury (SCI) model rats, researchers found that polydatin, a component of resveratrol, reduced oxidative stress and inhibited microglia apoptosis in rat brain by enhancing Nrf2/HO-1 signaling [129]. They also reported that polydatin upregulated Nrf2 activity in LPS-stimulated BV2 cells and knockout Nrf2 in BV2 cells increased oxidative stress and cell apoptosis [129]. Moreover, the activation of Nrf2 was demonstrated to prevent NLRP3 inflammasome activation induced by LPS in murine microglia as well as in the hippocampus of mice with depressive-like behavior (DLB) [130]. Accumulating evidence suggests DOR is a critical regulator of Nrf2 signaling in several neurological models. For instance, SNC-80, a DOR agonist, was reported to upregulate Nrf2 and HO-1 protein and protect mice against MPTP injury in vivo [15]. Hypoxia/ischemia stress can trigger an increase of Nrf2 nuclear protein and its downstream proteins such as HO-1 and NQO-1 to inhibit neuroinflammation. Cao et al. reported that DOR activation further amplified such protective reaction in the cortex of rats subjected to hypoxia/ischemia by enhancing the expression and translocation of Nrf2 after hypoxia/ischemia. The administration of DOR antagonist naltrindole can block the DOR’s effects on Nrf2 [21, 75]. Although there is no direct evidence proving that DOR regulates Nrf2 signaling in microglia, it is definitive that DOR activation modulates oxidative stress and inhibits microglial pro-inflammatory responses in LPS/ hypoxic injury [22, 100], which may relate to DOR’s regulation of Nrf2 signaling.

### 6.4 Caspases

Experiments performed in the BV2 microglia cell model showed that DOR activation attenuated LPS-induced inflammation and cell apoptosis by inhibiting MAPK/caspase-3 pathway [68]. Mounting evidence supports that caspases are tightly regulated by DOR under a series of stresses [12, 13]. Orderly activation of the caspases cascade is known for its initiation of apoptotic cell death. However, caspases regulated microglia activation through a PKC-δ dependent way has been well described [131]. Bruguillos et al. found that several stimuli could activate caspase 3/7 and caspase 8 in microglia without triggering cell apoptosis in vitro and in vivo. Knocking down or inhibiting each of these caspases attenuated microglial activation and reduced neurotoxicity [131]. The phenomenon was investigated in the model of PD and AD in vivo, suggesting a DOR-caspases-mediated anti-microglia activation in neurogenerative diseases.

Combined with these observations, we can regard the opioids mediated pro-inflammatory effects as an opioid receptor-independent interaction on microglia, while the binding of opioids to opioid receptors, especially the DOR-specific agonist with DOR, is another story in microglia (Figure 2). Although the exact role of DOR in microglia and its signaling pathways in microglia remains to be investigated, increasing evidence points out the potential signaling crosstalk between DOR and microglia in neuroinflammation as Figure 2 shows.

## 7. Conclusion and Perspectives

Excessive activation of microglia or microglia dysfunction is implicated in several neurological diseases. The neuroinflammatory responses of microglia at different pathological stages differentially impact the disease progression. There are several aspects through which microglia affect neuroinflammation, including releasing the inflammatory cytokines, chemokines, and superoxide, which impair mitochondrial integrity and promote neuron apoptosis. Production of inflammatory cytokines and chemokines can also transform the astrocytes into the toxic phenotype and disrupt the BBB, amplifying the neuroinflammatory attack on the brain. The complex mechanisms underlying microglia-mediated neuroinflammatory regulations are closely associated with microglial dynamic phenotype transformation, its interactions with several receptors, and the multiple pathways involved in its immune responses.

Limited literature documented the potential role of opioid receptors in neuroinflammation and their expression in microglia. The function of opioids on microglia are typically linked to pain modulation. Some studies reported that morphine is positively related to microglial activation, but this may be due to its binding with TLR4, instead of MOR or other opioid receptors, in microglia. Early studies have well demonstrated that DOR activation protects neurons at multiple levels under different stresses, but the studies regarding the function and the action mode of DOR in microglia are just getting started. Recent research shows the potential linkage between DOR and microglia in the regulation of neuroinflammation. DOR activation was reported to suppress microglia polarization and thus inhibited the release of several inflammatory mediators in LPS or hypoxia-exposed cultured cells and animal models [22, 99, 100]. Moreover, the function of microglial DOR may lie beyond this. Our recent sequencing analysis showed that knocking-down DOR in BV2 cells largely affected the genes responsible for protein binding, microglia locomotion, adhesion, and microglial responses to various stimuli. DOR knockdown also interfered with the signaling of other intracellular molecules including extracellular matrix (ECM) receptor, MAPK, and NOD-like receptor and thus altered the course of microglial inflammation (Yuan Xu et al. unpublished data). Moreover, DOR regulates several ions, receptors, neurotransmitters, and pathways involved in microglia as we previously studied [8], which determines its complex role in microglial dynamics. Until now, the signaling transduction of DOR in microglia, its relationship with microglial cellular compartments, and its detailed mechanism in modulating microglial morphology, gene expression, polarization, and function are almost unknown. More research is required to validate the complete DOR signaling transduction in microglia, and more investigations are needed to evaluate the function of DOR in microglia.

Another important question is whether DOR affects the same signaling cascades in microglia as in neurons? Current progress in DOR research implies that DOR activation potentially inhibits p38 and caspases in microglia as it does in neurons. However, there are some differences between the appearance of DOR in microglia versus neurons. For instance, it is reported that DOR activation down-regulated ERK phosphorylation in BV2 cells to protect cells against LPS injury [100], while in neurons subject to acute hypoxic or MPP^+^ stress, DOR activation enhanced ERK phosphorylation instead [13]. Moreover, our studies in neuron-like cell lines, the highly differentiated PC12 cells, showed that MPP^+^ and hypoxia-induced a decrease in Akt phosphorylation, while the application of DOR-specific agonist UFP-512 activated Akt signaling both under MPP^+^ insults and hypoxic insults [13]. Since DOR can trigger the activation of the PI3K/Akt pathway, and PI3K/Akt/ mTOR is required for microglia activation, it seems that DOR activation also plays a positive role in promoting microglia polarization. However, our recent studies showed the opposite effects of DOR in regulating microglia activation [22]. Knocking down DOR in BV2 cells promoted BV2 excessive activations in both M1 and M2 directions with a significant increase in both M1 marker and M2 marker [22]. The reason for this contradiction is unknown and may be associated with more complex mechanisms underlying the interplay between DOR and microglia.

Taken together, in this review, we pointed out the critical role of microglia in regulating neuroinflammation and its related diseases. We summarized the current progress of DOR research in microglia: its expression pattern, pharmacological effects on neuroinflammation, and potential signaling transduction in microglia. Current research revealed a beneficial tendency in slowing down cerebral disease progress with DOR activation, especially the age-related neurological disorders. Furthermore, in future projects, we will further explore DOR’s other functions in microglia, compare the similarities and differences of DOR’s method of action and its signaling transduction in microglia versus neurons to better understand it. Clues have indicated that there are discrepancies between human and mouse microglia signatures in neurodegeneration [35]. Therefore, how to obtain human microglia and better monitor human pathological state is another crucial problem in our way to study DOR.
